# The Physical Therapy Compact: From Development to Implementation

**DOI:** 10.5195/ijt.2017.6237

**Published:** 2017-11-20

**Authors:** LESLIE ADRIAN

**Affiliations:** FEDERATION OF STATE BOARDS OF PHYSICAL THERAPY, ALEXANDRIA, VA, USA

**Keywords:** Compact, Physical therapy, Telehealth

## Abstract

The Federation of State Boards of Physical Therapy (FSBPT) began the process of implementing the Physical Therapy Compact (PTC) in 2010 with a delegate assembly motion. An interstate compact is an agreement between states to enact legislation and enter into a contract for a specific, limited purpose or to address a particular policy issue. An interstate compact benefits patients, consumers, and licensees by addressing workforce concerns improving licensure portability from state-to-state; facilitating short-term mobility and telehealth consultation for physical therapists. FSBPT formed strategic partnerships with the American Physical Therapy Association (APTA) and Council for State Governments’ (CSG) to develop and implement the PTC. From April 2014-present, FSBPT followed the CSG recommended stages of compact development: Advisory, Drafting, Education, Enactment, and Transition. The enactment phase began once the threshold number of ten states required in legislation passed the compact bill in 2017. FSBPT anticipates the compact will be fully operational and licensees will be able to take advantage of the compact by mid-2018.

The Federation of State Boards of Physical Therapy (FSBPT) is a membership organization comprised of the 53 jurisdictional licensing boards regulating physical therapy in the United States. The FSBPT mission is to protect the public by providing service and leadership that promote safe and competent physical therapy practice. At its 2010 Annual Meeting, the FSBPT Delegate Assembly passed a motion to explore the feasibility of establishing a multi-state compact for physical therapy licensure. This motion originated from the acknowledgement that a multi-state licensure compact for physical therapy would increase public protection by positively impacting access to care and facilitating the sharing of disciplinary information. A compact benefits patients, consumers, and licensees by addressing workforce concerns improving licensure portability from state-to-state; facilitating short-term mobility and telehealth consultation for physical therapists.

Many organizations are currently having discussions concerning alleviating unnecessary barriers to licensure portability among states. Federal and state governments, the National Governors Association, consumer groups, the American Telemedicine Association, professional associations, and regulatory bodies have all looked into how to expedite the mobility of professionals while still maintaining high standards and protecting the public. The diversity of these groups leads to different end-goals in the improved mobility of health professionals, but overall most groups agree improved portability is one way to address workforce needs and improve the public’s access to health care services.

Although there have been numerous changes in the health care practice environment, until the Nurse Licensure Compact was introduced in the late 1990s, there had been little in the way of innovation in the fundamental processes of health professional licensure. Although improvements such as on-line processing and electronic renewals have been implemented, the single-state system of professional licensure remained as the preferred model of regulation in most states. However, in the last 5 years, there has been a lot of interest and momentum in alternative licensing models, including compacts for Physicians (Interstate Medical Licensure Compact; IMLC), Psychologists (Psychology Interjurisdictional Compact; PSYPACT), EMS providers (Recognition of EMS Personnel Licensure Interstate CompAct; REPLICA), the revision of the Nursing Licensure Compact (eNLC), and of course the Physical Therapy Compact (PTC).

An interstate compact is an agreement between states to enact legislation and enter into a contract for a specific, limited purpose or address a particular policy issue. Interstate compacts should not be entered into casually by a state. Compact agreements are unique in their duality as statute and contract. Each state must understand the implications of entering into a contract and must meet the terms required of all compact members.

A key component of compacts is the uniformity of legislative language from state to state. To enact a compact, the legislative language be the same from state to state, thus model legislative language is provided to states interested in entering the compact. In order to join the compact in question, the state must pass legislative language that in no way materially changes the compact in order to implement the compact. Any new state interested in joining the compact must have any proposed changes to language of the model legislation reviewed by the Commission attorneys to see if the change(s) qualify as a material difference. If so, the parties involved must understand that the bill as written may not be implemented, even if it has passed the legislature.

FSBPT followed the Council for State Governments’ (CSG) recommended steps to develop the PTC: Advisory, Drafting, Education, Enactment, and Transition. A compact regarding healthcare licensure is unique in that the initial interest will most likely be from the Board or professional association rather than originating with a legislator. FSBPT made the decision early on to maintain transparency in all communications regarding the PTC with any interested parties and partner with the American Physical Therapy

Association (APTA) in the efforts to develop a compact. The buy-in and support of the professional association was critical for a successful educational campaign reaching out to their leadership and membership regarding the PTC. According to CSG, an organization interested in a compact should begin by convening an advisory group of key stakeholders to take a high level look at the key elements and challenges of an interstate compact. The Advisory Group typically meets one or two times, with their work culminating in a set of recommendations as to whether or not to move forward with a compact and what the final compact product should look like. A misconception is that compacts all look alike; in fact the reality is quite the opposite. Compacts can, and should, be tailored to the needs of the profession to which it pertains. If the advisory task force determines a compact is feasible, they are also tasked with determining the high level specifics of how the compact will operate. These high level specifics will be used by the drafting team to make sure the final language remains true to the spirit of the advisory team.

The PTC Advisory Task Force met during the months of April–July of 2014. FSBPT cast a wide net when choosing task force members. The rationale was that the broadest representation of key stakeholders representing different interests and geographical areas would yield the best discussions and culminate in a recommendation that would have the best chance of success. The task force was composed of state board members, board administrators, a state senator, physical therapists, lawyers, and professional association (American Physical Therapy Association (APTA)) representatives, with representatives from CSG providing technical assistance. The advisory task force assembled by FSBPT examined the concept of a compact for physical therapy broadly. The task force recommended moving forward with the drafting of the PTC and culminated in suggestions for the final compact product.

The next step is to assemble a smaller, more focused group of 5–8 individuals to serve on the Drafting Team. FSBPT chose to retain a core of individuals from the advisory task force and supplemented with qualified individuals to bring an unbiased perspective. The drafting team is responsible to build upon the work of the Advisory Group to create the legislative language of a draft compact. This draft comment should be circulated for wide ranging input and comment from stakeholders in all areas, including the public. After revision, the draft compact would go back to the advisory group for final review and approval. The PTC Drafting Team met January–April 2015, tasked with actually drafting the statutory language of the PTC. CSG again provided technical assistance and support with the drafting of the statutory language. The drafting team crafted the language based on the recommendations of the advisory group, as well as concepts and issues that came up during their own discussions. The PTC draft document was released to a broad audience for a public comment period in May 2015. After receiving comments, the drafting team debated suggested changes and developed the final product on October 6, 2015.

The education phase begins immediately following the finalizing of model legislative language. Once the interstate compact is drafted and approved, the states are free to introduce legislation to implement the compact. FSBPT entered into an official partnership with CSG to provide state-by-state technical assistance and education before and during state legislative sessions. Educational efforts will continue after enactment to maximize membership in the PTC. Education of physical therapy licensing boards, licensees, and legislators is paramount to the success of the PTC. The professional association, the Board members, the legislature, and any overarching Boards will need to be educated about the terms and advantages of the compact. Once the PTC language was completed, FSBPT began an enthusiastic campaign to educate the membership regarding the compact. During the same time, APTA began with an educational push of their own, and both organizations worked closely to maintain a consistent message.

The enactment phase begins once the threshold number of states required in legislation passes the compact bill. The PTC required ten states to pass compact legislation prior to the compact “going live.” FSBPT staff believed in late 2015, based on the interest communicated by a number of states, that by the conclusion of the 2017 legislative session, the PTC should pass in ten states and be enacted. In 2016 the first four states adopted the PTC: Oregon, Tennessee, Arizona and Missouri. Ten more states followed suit in 2017; Table 1 below shows the effective dates of PTC legislation in member states.

The final stage, transition, occurs post-enactment. Although the PTC came into effect in April 2017 when the statute was enacted into law in the tenth member state, the PTC will not be operational for licensees until the Physical Therapy Compact Commission finalizes some standard start-up activities, including the first Commission meetings where the member states meet to discuss development of rules, bylaws, etc., by which the PTC will be governed. Finally, when the compact body is able to run independently, licensees will be able to take advantage of the benefits of the PTC. This is when licensees will begin to work in states other than their home-license state with a Compact Privilege rather than a license. FSBPT anticipates licensees will be able to take advantage of the compact by mid-2018.

## Figures and Tables

**Table 1:** Effective Dates of Physical Therapy Compact (PTC) Legislation in Member States

State	Bill #	Effective Date
Oregon	SB 1504	3/3/2016
Tennessee	SB 2368	4/14/2016
Arizona	HB 2504	5/17/2016
Missouri	HB 1816	8/28/2016
North Dakota	HB 1157	3/13/2017
Utah	SB 248	5/9/2017
Kentucky	HB 227	6/29/2017
Mississippi	HB 309	7/1/2017
Washington	HB 1278	7/23/2017
Montana	HB 105	10/1/2017
Colorado	HB17–1057	5/10/2017
North Carolina	HB 57	10/1/2017
Texas	SB 317	9/1/2017
New Hampshire	SB 212	7/1/2017
